# Intermediate-Temperature Tensile Behavior of a Hot-Rolled Mg-Li-Al-Cd-Zn Alloy

**DOI:** 10.3390/ma15051686

**Published:** 2022-02-24

**Authors:** Lunyong Zhang, Yongjiang Huang, Ming Wu, Chao Xu, Zhiliang Ning, Fuyang Cao, Jianfei Sun

**Affiliations:** 1 School of Materials Science and Engineering, Harbin Institute of Technology, Harbin 150001, China; zhangly@hit.edu.cn (L.Z.); yjhuang@hit.edu.cn (Y.H.); wuming@c-wst.com (M.W.); cxu@hit.edu.cn (C.X.); caofuyang@hit.edu.cn (F.C.); jfsun@hit.edu.cn (J.S.); 2 National Key Laboratory for Precision Hot Processing of Metals, Harbin Institute of Technology, Harbin 150001, China; 3 Western Superconducting Technologies Co., Ltd., Xi’an 710018, China; 4 Ctr Anal & Measurement, Harbin Institute of Technology, Harbin 150001, China

**Keywords:** Mg-Li-Al-Cd-Zn alloy, mechanical property, medium-temperature, microstructure

## Abstract

Developing light structure materials that work stably at elevated temperatures is a long-standing challenge for many application fields, particularly in the development of aerospace equipment. Zn/Cd alloying elements were prospected to improve the stability of the lightest Mg-Li based alloys; however, little is known about the intermediate-temperature mechanical properties of such alloys. The present work investigated the tensile behaviors of a cold-rolled Mg-Li-Al-Cd-Zn alloy in a temperature range of 30–150 °C. The results indicate that the alloy can host a tensile strength σ_UTS_ of 108~121 MPa, a yield strength σ_YP_ of 97~109 MPa and elongation *ε*_B_ of 14–15 % at 150 °C, dependent on the tensile direction. The mechanical properties intensively are modulated by temperature through the competition between work hardening and softening. Work hardening due to dislocation blocking by the precipitated MgLi_2_X phase dominated the deformation at low temperatures, while softening that resulted from dynamic recrystallization was the main effect at high temperatures. Correspondingly, a quasi-cleavage mechanism dominated the fracture at temperatures near room temperature, and microvoid coalescence worked at high temperatures above 100 °C. Our results offer a new experimental understanding of the elevated-temperature mechanical behaviors of Mg-Li alloys and will advance the development of new light magnesium alloys with high stability.

## 1. Introduction

As the lightest structural metal material, Mg-Li alloys are expected to be widely used in the aerospace, health care, automobile and electronic industries because of their low density, high specific strength and elastic modulus, excellent formability and damping property [1,2,3,4]. However, the relatively low strength, poor corrosion resistance and limited thermal stability of binary Mg-Li alloys severely hinder their practical applications [5,6]. It is well documented that Mg-Li alloys with a hexagonal closed-packing structure phase (hcp-α) have a moderate strength and a limited formability, while the body-centered cubic structure phase (bcc-β) Mg-Li alloy demonstrates an excellent workability but notably lower strength [7,8]. Thus, the duplex phase (hcp-α + bcc-β) strategy was often adopted to achieve good comprehensive mechanical properties of Mg-Li alloys, leading to promising results for structural materials [7,8], which inspired the development of a series of commercial duplex phase Mg-Li based alloys, typically the MA21 (ИMB2) alloy with alloying elements of Li (7.5–9.0 wt.%), Al (4.5–5.3 wt.%), Zn (1.0–2.0 wt.%), Cd (4.0–5.0 wt.%) and minor amount of Mn (0.03–0.1 wt.%) as well as Ce (0.01–0.05 wt.%). The room temperature mechanical performances of MA21 reach *σ*_B_ = 200–260 MPa, *σ*_0.2_ = 130–220 MPa and δ = 6–20 % [9]. 

A big challenge for Mg-Li alloy application is the low stability due to the high mobility of lithium atoms and vacancies even under room temperature, which could be relieved by the addition of Zn and Cd in the MA21 alloy [9]. Thus, the MA21 alloy has the potential of working at the elevated temperatures encountered by aerospace shuttles. It is thus necessary to investigate the elevated temperature mechanical properties of this material. About forty years ago, Koryagin et al. reported that the mechanical behaviors of an as cast MA21 alloy during long term aging or at raised temperatures is mainly confined by a complicated phase evolution process, the transition from the metastable phase θ to an equilibrium phase AlLi and also the solution and coagulation of precipitated hardening phases MgLi_2_Al and AlLi [10]. Furthermore, about thirty years ago, Kaibyshev reported the superplastic deformation behaviors of as cast MA21 alloys in a temperature regime of 350–500 °C (0.75–0.93 Tm, Tm is absolute melting point in Kelvin which is ~833 K for the present MA21 alloy) [11]. The flow stress was decreased until the temperature increased to around 450 °C where the relative elongation reached a maximum of 450%. Strong stain rate dependence of the flow stress was also reported for the MA21 alloy [11]. Increasing deformation temperatures would cause lower flow stress and strain. The dynamic recrystallization process dominated the deformation behaviors [11]. 

These studies brought us a general impression of what would happen for an MA21 alloy working at temperatures near its melting point. The complicated phase evolution process builds the core for understanding the high temperature mechanical behaviors. In practice, three temperature regimes were often subdivided for discussing the temperature driven mechanical behaviors, i.e., high-temperature *T* > 0.6*T*_m_, intermediate-temperature 0.3*T*_m_ < *T* < 0.6*T*_m_, and low-temperature *T* < 0.3*T*_m_ [12]. Much attention is conventionally paid to the high temperature regime, as has been done for the present MA21 alloy. 

The intermediate-temperature mechanics are also interesting because it is generally believed that the dominating mechanism of a metal deformation at different temperature regimes would be distinct [13] and some application conditions of Mg-Li alloys are covered by the intermediate-temperature regime [14]. We recently investigated the creep behaviors of an as-rolled MA21 alloy (Mg-7.9Li-5.3Cd-4.5Al) at temperatures covering the regime of ~0.3–0.5 *T*_m_ (~30–150 °C). It was revealed that the precipitated MgLi_2_X (θ-phase, X = Al, Zn, Cd) would prohibit dislocation motions and thus improve the creep resistance and extensive atom diffusion of Al. Furthermore, Cd elements act as the drag force source dominating the dislocation slip process for the creep behaviors at intermediate-temperatures and low stresses [15]. The sample could sustain the stress of several megapascals (MPa) for hours. In general, temperature tunes the microstructure of MA21 alloys and dramatically modifies the mechanical performances. 

So far, it is less known about the tensile performances of MA21 alloy at intermediate- temperature regime, this will be investigated in the present work in temperature range ~0.3–0.5 *T*_m_. Our results revealed the tensile deformation behaviors of an as-rolled MA21 alloy was controlled by the competition of work hardening and softening. Work hardening dominates at low temperature and softening works at high temperature. The precipitated MgLi_2_X with X = Al, Zn or Cd phase plays a great role as the microscale structure controlling the competition of work hardening and softening. Correspondingly, quasi-cleavage mode dominates the fracture of the specimens at low temperature, which transisted into microvoid coalescence mechanism at high temperature. These finding fills the blank of understanding of the intermediate-temperature mechanical behaviors of MA21 alloy, would prompt the composition design and mechanical properties modification of magnesium alloys.

## 2. Materials and Methods

**Figure 1:**
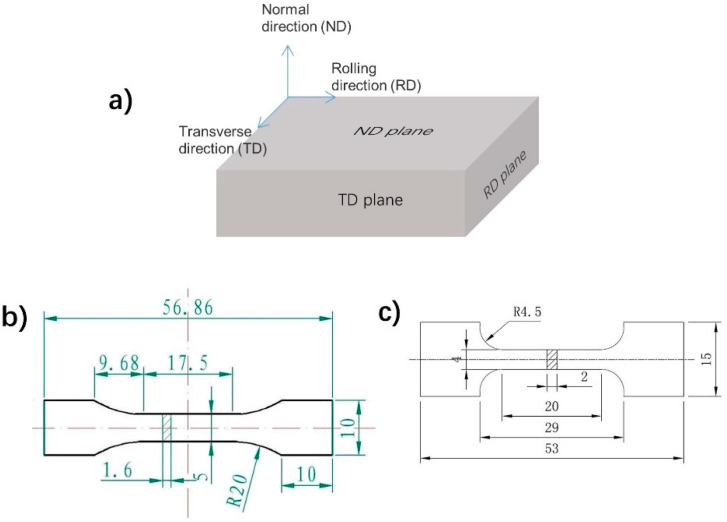
Schematic geometries of (**a**) the as-received heat-rolled MA21 alloy plates, (**b**) the specimens for room temperature tensile tests and (**c**) the specimens for tensile tests at elevated temperatures.

The Zhengzhou Light Metal Research Institute, China supplied the as heat-rolled MA21 alloy plate for the present work (as seen Figure 1a for the rolling geometry). Its composition is Mg-7.99Li-5.3Cd-4.57Al in wt.% with minor elements Zn (0.8 wt.%), Mn (0.05 wt.%) and Ce (0.03 wt.%). Through a rolling process with the geometry shown in [15], the plates, which were originally 60 mm thick, became 24 mm thick. Tensile tests at room temperature and elevated temperatures were carried out with a tensile rate of 2mm/min, according to ASTM: E20900. The specimen geometries are shown in Figure 1b,c, respectively. Three repetitions were carried out at each temperature. An Olympus PMG3 type optical microscopy (OM) was adopted to observe the metallographic microstructures. The metallographic samples were cut from the specimens followed by polishing and chemical etching for 5–20 s in an alcohol solution containing nitric acid (2% in volume fraction). A Quanta 200FEG type scanning electronic microscopy (SEM) was used to image fracture the microstructure. A Talos F200X transmission electronic microscopy (TEM) was also used to characterize the microstructure by using the high angle annular dark field mode (*HAADF*). Electron back-scatter(ed) diffraction (EBSD) equipped on a HELIOS NanoLab 600i SEM was used to analyze texture. The EBSD signal was collected with the Oxford-HKL system at a voltage of 20 KV and a tilting angle of 70°. The step size was 10 μm. About 1000 grains were analyzed for each condition. Cubic plates that were 12 × 10 × 8 mm^3^ were extracted from the specimen surface and then polished for texture analysis. 

The tensile tests at room temperature were carried out on an Instron5569 electromechanical test machine, and an Instron 5500R machine was used to test the tensile properties at elevated temperatures. The tests were performed along the rolling direction (RD) and transverse direction (TD), respectively, at RT, 50 °C, 75 °C, 100 °C, 125 °C and 150 °C. All tests were repeated three times. The sample surface planes are thus defined by their normal directions as RD, TD and ND planes, where ND is the direction perpendicular to the rolling surface. 

## 3. Results

**Figure 2:**
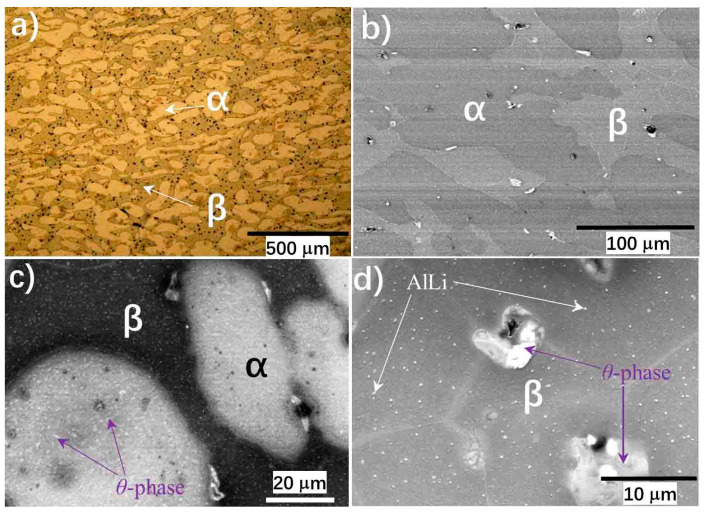
Microstructures of the as-rolled MA21 alloy plates. (**a**,**b**) show the α + β duplex phase structure observed by OM metallographic and TEM, respectively. (**c**,**d**) display the fine phase structure observed by back-scattered electrons model of SEM in the α and β phase, respectively.

Figure 2 demonstrates the microstructures of the as-rolled MA21 plates at different scales. Typical duplex phases were confirmed by the OM metallographic analysis (Figure 2a) and also by TEM at a low magnification scale (Figure 2b). The two phases were assigned as the α-Mg phase and β-Li phase by a combination of the XRD and energy dispersive spectrum (EDS) analysis results (see our previous work [15] for detail) and the reported phase composition of the MA21 alloy [9]. At a finer scale, bright precipitated phases can be observed in both the α-Mg phase (Figure 2c) and β-Li phase (Figure 2d). According to the XRD and EDS results reported in [15], the bright precipitated phase in the α-Mg phase is the θ-phase of composition as MgLi_2_X with X = Al, Zn or Cd [11], the particle-like bright phase in the β-Li phase is the AlLi phase and the larger cluster-like phase at β-Li phase boundary is the θ-phase. 

**Figure 3:**
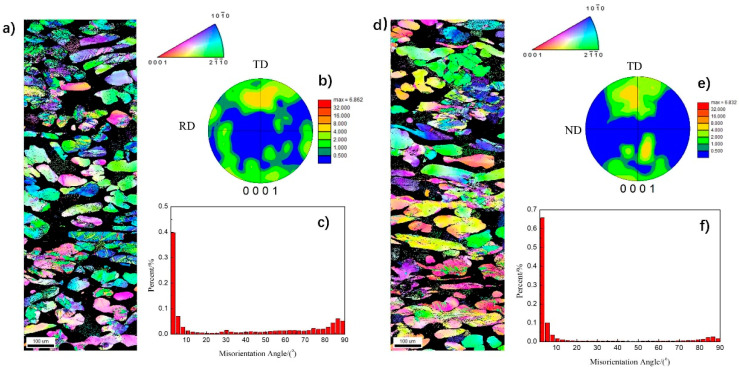
EBSD analysis results of the as-rolled plates. (**a**–**c**) demonstrate the EBSD image observed on the ND plane, the corresponding {0001}-polar figure and grain orientation difference diagram, respectively. (**d**–**f**) shows results for the RD plane.

Figure 3 shows the texture analysis result by the EBSD collected on the ND plane (Figure 3a–c) and the RD plane (Figure 3d–f) of the as-rolled plates. The grain size in the ND plane is therefore determined as 37.7 μm in average. Most grains in the ND plane are aligned with the [2$\bar{1}$$\bar{1}$0] or [10 $\bar{1}$ 0] crystallographic orientation towards the normal direction (ND) or deviate from it with a small angle (Figure 3a). The {0001}-polar figure of the ND plane (Figure 3b) indicates that the [0001] orientation of grains points to the transverse direction (TD) and bears a 45° angle with respect to the ND plane. Figure 3c shows the statistic diagram of the grain orientation differences. The grain orientation differences for most grains are smaller than 15°. Grains with orientation angle differences smaller than 5° reach 40%, suggesting the grain boundary is dominated by the low-angle boundary. This kind of small angle boundary could be the result of a dislocation slip during the rolling process. Figure 3c also indicates that there are grains with orientation difference larger than 80°, almost to 5%, which corresponds to high-angle boundaries. On the RD plane, Figure 3d indicates that the grains are aligned without preferred orientations. However, the {0001}- polar figure reveals a texture with the [0001] orientation towards the transverse direction (TD). With respect to the ND plane, the grain boundaries in the RD plane are mainly low-angle boundaries, as suggested by the grain orientation difference diagram (Figure 3e). The TD plane data have been reported in previous work [15] and so are not shown here. Generally, the grain texture on this plane is stronger than that on the RD plane. Most grains have their [0001] orientation aligned to the transverse direction (TD) [15], which is consistent with the conclusion suggested by the {0001}-polar figure of the ND plane (Figure 3b).

**Figure 4:**
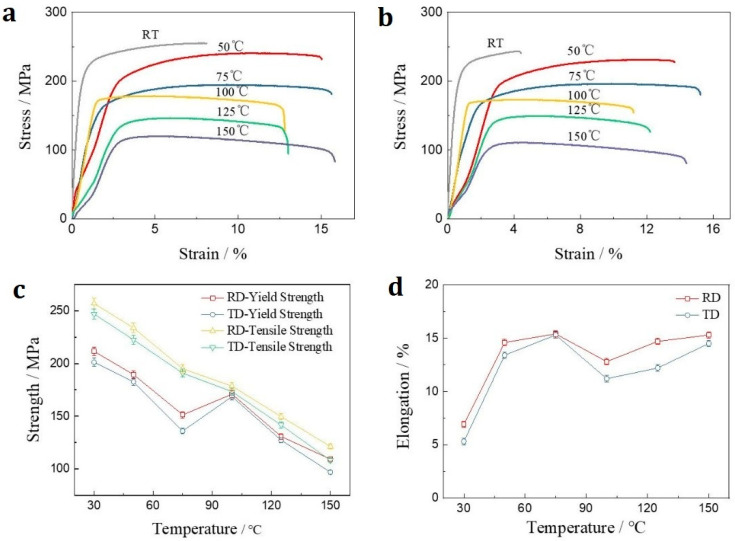
Tensile behaviors at elevated temperatures. (**a**,**b**) are the engineering stress–strain curves measured from the sample with force loading along RD and TD, respectively. (**c**,**d**) respectively demonstrate the evolution of yield strength, tensile strength and elongation with temperature extracted from (**a**,**b**).

Taking into account that the grains prefer to align their [0001] towards the TD, the tensile tests were carried out along the RD and TD at varied temperatures. The strain–stress curves in Figure 4a,b clearly suggest plastic deformation behavior at all measured temperatures. At temperatures lower than 75 °C, remarkable work hardening occurred for the specimen regardless of the force loading direction, which disappeared at higher temperatures where strain softening occurred, as previously reported in the temperature range of 0.75–0.93 *T*_m_ for MA21 alloys [11]. There should be a transition regime of 75–100 °C in which the work hardening and the softening compete extensively. Such work hardening at low temperatures and softening at higher temperatures was also observed at other magnesium alloys, for example, the AZ31 alloy suffered tension [16]. Temperature exerts prominent effects on the mechanical properties of MA21 alloys, following the common feature of magnesium alloys. 

Figure 4c,d and Table 1 summarize the mechanical property parameters of the specimen at varied temperatures. The force loading direction did not affect the parameters seriously. The tensile strength *σ*_UTS_ and yield strength *σ*_YP_ reached about 247–257 MPa and 200–211 MPa at room temperature (30 °C), respectively, and were still kept at a level of about 108–121 MPa and 97–109 MPa at 150 °C, respectively, dependent on the loading direction, even though they decreased with the temperature rise. It is interesting that an enhancement jump appeared for the yield strength at the temperature transition from 75 °C to 100 °C where the work hardening and the strain softening compete extensively, and then the yield strength decreased stably with temperature increasing, as seen in Figure 4c, suggesting that the enhancement jump of yield strength does occur. The microscale event occurred in the temperature range of 75–100 °C to induce the transition of the deformation mechanism as well as to trigger the jump; however, this did not cause the tensile strength to jump. The mechanism should be attributed to the precipitation of the MgLi_2_Al phase from the matrix when the temperature reached around 100 °C, which is the incubation temperature of the MgLi_2_Al phase. The dispersion distribution of the MgLi_2_Al phase could block the dislocation motion and induce the strengthening effect [15]. Moreover, Figure 3d displays a general trend of elongation evolution. The elongation increased from ~5% to ~15% when the temperature was within a narrow window from RT to about 75 °C. Then, the elongation did not demonstrate dramatic changes when the temperature was increased further. Even an elongation fluctuation was still distinguished there. The elongation first decreased at temperatures from 75 °C to 100 °C and then increased. This temperature window associated with elongation suppression was consistent with the temperature regime where the yield strength enhancement jump appears, thus suggesting that this elongation evolution has the same mechanism as the yield strength evolution observed in Figure 4c. Both are the result of the completion between the precipitation strengthening of MgLi_2_Al and the strain softening with the rise in temperature. At the temperature region around 100 °C, the rapid precipitation of the MgLi_2_Al phase distinctly prompts the strengthening effect, which would be kept almost constant at a higher temperature regime. Meanwhile, the softening effect is enhanced when the temperature increases. Thus, a jump in the yield strength and a drop in elongation were observed. 

**Figure 5:**
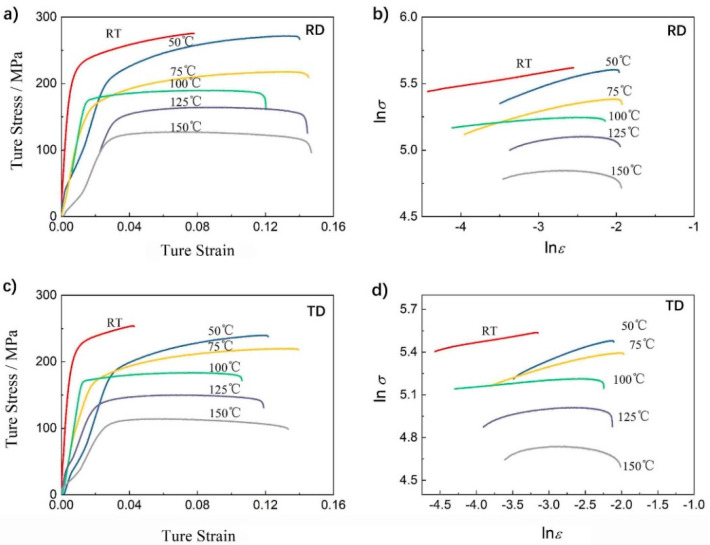
Work hardening exponent extraction. (**a**,**c**) are the true *σ* vs. *ε* data for the tensile tests along RD and TD, respectively. (**b**,**d**) are the ln*σ* vs. ln*ε* curves for work hardening exponent extraction based on (**a**) and (**c**), respectively.

The work hardening exponent *n* was often adopted to measure how quickly a material gains strength during deformation. A relationship is held between true stress *σ* and true strain *ε* through n as the following [17].

(1) $$\sigma =k\varepsilon^{n}$$

where *n* and *k* are constants. Figure 5a,c exhibits the *σ* vs. *ε* data for the tensile tests along RD and TD, respectively. Accordingly, the hardening exponent n could be extracted through logarithming the Equation (1). This was done in Figure 4b,d, respectively, for the *σ* vs. *ε* data of RD (Figure 5a) and TD (Figure 5c).

**Figure 6:**
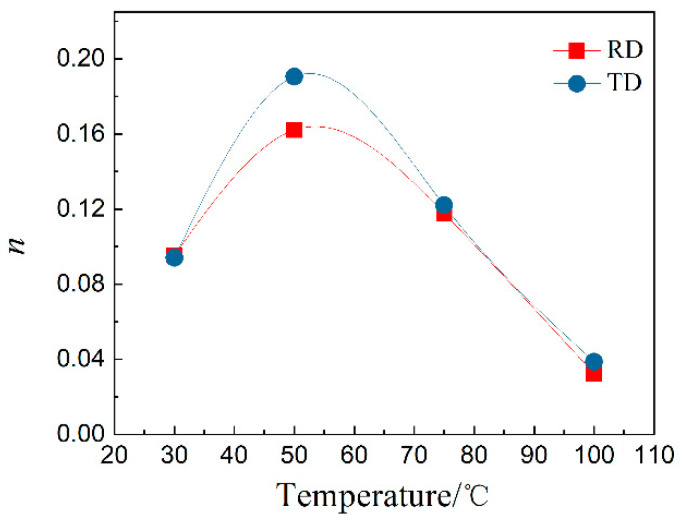
Temperature dependence of work hardening exponent.

Thus, the exponent n was extracted and shown in Figure 6. The exponent is around 0.09 at room temperature and increased to ~0.16 or ~0.20 at 50 °C dependent on the force loading direction, then decreased when the temperature increased. The exponent is around 0.04 at 100 °C. The exponent at 150 °C cannot be extracted owing to the strong nonlinearity of the ln*σ* vs. ln*ε* curve at 150 °C. Thus, temperature clearly affects the tensile behavior of the MA21 alloy specimen. 

Meanwhile, it was shown from Figure 4c,d that the tensile performance is slightly better when the force loading is along the RD, which showed higher tensile strength, yield strength and larger elongation. In contrast, the work hardening exponent for the test along the RD is slightly smaller than that along the TD except at room temperature, see Figure 6. The weak texture in the as-rolled plate (Figure 3) should be responsible for such weak anisotropy of mechanical properties. 

**Figure 7:**
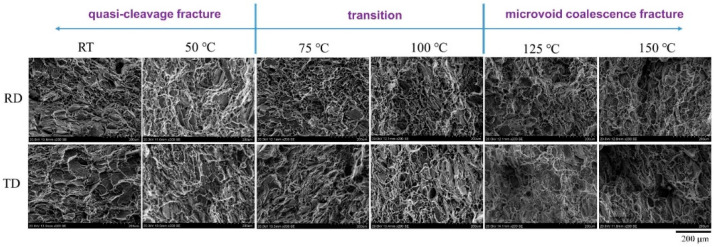
Fracture surface morphology.

**Figure 8:**
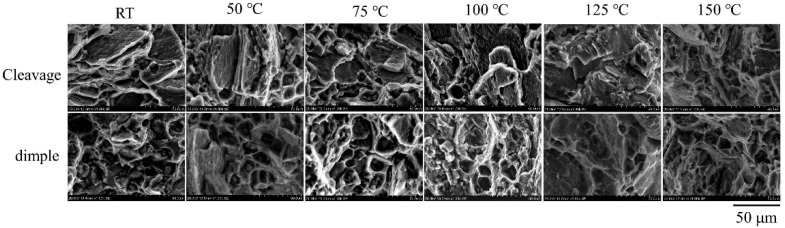
Morphology evolution with temperature of the cleavage structure and the dimple structure.

Figure 7 further demonstrates the fracture surface morphologies. No distinct morphology differences can be seen for the fracture surfaces of the RD and TD samples. Cleaved planes were the main feature of the fracture morphology at low temperature RT and 50 °C and dimple is the main morphology feature at temperatures of 125 and 150 °C. The transition regime is almost in the temperature range of 75–100 °C, where the fracture surface contains large numbers of both cleaved planes and dimples. It was shown that there are river-like patterns on the cleaved plane surface (upper panel in Figure 8). The pattern density increases with the temperature until reaching 125 °C. Above 125 °C, the river-like pattern almost disappeared. Simultaneously, the size of the dimples increases with the temperature (lower panel in Figure 8). Meanwhile, we noted that there are particles in the dimples, particularly at low temperatures. The particles became gradually diminished as the temperature rose and disappeared at 125 °C. Accordingly, the fracture surface morphology evolution behaviors suggest a quasi-cleavage fracture mechanism at low temperatures at RT and 50 °C and a microvoid coalescence mechanism at 125 °C and 150 °C. In the temperature range of 75–100 °C, the fracture mechanism transition proceeded.

## 4. Discussion

The above results revealed a moderate plasticity of the as-rolled MA21 alloy at temperatures lower than 0.5 *T*_m_, and the tensile deformation in the plastic stage was controlled by work hardening until the temperature rose to the window of 75–100 °C, above which the strain softening emerged to dominate the deformation. A transition went through the temperature window in which the work hardening and strain softening come to a state approaching balance. A general model connecting the macroscopic deformation process to the microscale dislocation evolution indicates such competition because stress is believed to be proportional to the square root of dislocation density *ρ*, as shown in the following [16]:

(2) $$\frac{d\rho }{d\varepsilon }={\left(\frac{d\rho }{d\varepsilon }\right)}_{h}-{\left(\frac{d\rho }{d\varepsilon }\right)}_{r}$$

The first term on the right hand frames the dislocation accumulation responsible for the work hardening, while the second one accounts for the dislocation annihilation associated with the strain softening. It has been shown in our previous studies of creep behaviors that the precipitate θ phase in the α-Mg and β-Li matrix (Figure 2c,d) would seriously block the dislocation motion and cause dislocation storage at low temperatures [15], which increases the dislocation density and should be the microscale mechanism inducing the observed work hardening. 

As for the strain softening, the steady flow stress state shown on the strain–stress curves around 100 °C is an obvious dynamic recrystallization characteristic [18]; that is to say, dynamic recrystallization induced the strain softening in the current specimens. In fact, obvious grain rotation was noted in the creep tests of the studied MA21 alloy [15]. On the other hand, it was shown that the θ phase in the α-Mg is partially decomposed only at high temperatures, for example, 125°C, which prompted the formation of a secondary phase of Al and Cd at the α/β phase boundary [15]. The decomposition of the θ phase was also hinted at in the present study by the gradual disappearance of particle-like matter in the dimples on the fracture surface as the temperature increased (lower panel of Figure 8). This would weaken the dislocation blocking center responsible for the work hardening and also prompt the softening to be come dominant. 

## 5. Conclusions

In summary, the present work investigated the tensile behaviors of an as-rolled MA21 alloy at a medium-temperature regime of 0.3–0.5 Tm to evaluate the elevated temperature mechanical performances of MA21 alloys. It was shown that the specimens performance well with tensile strength reaching ~250 MPa, a yield strength of ~200 MPa and an elongation of about 5% at room temperature. Temperature intensively modulated the tensile behaviors through the competition between working hardening due to dislocation blocking by the precipitated θ phase and strain softening supported by dynamic recrystallization. Increasing temperature decreased tensile strength and yield strength, but enhanced elongation. They approached ~100 MPa, ~100MPa and ~15%, respectively, at 150 °C. It was revealed that the quasi-cleavage mechanism dominated the fracture of the MA21 alloy specimens at temperatures near room temperature, and microvoid coalescence worked at temperatures above 100 °C.

## Figures and Tables

**Table 1:** Summary of the mechanical properties of the MA21 sample at different temperatures.

Temperature/°C	*σ*_YP_/MPa		*σ*_UTS_/MPa		*ε*_B_/%	
	RD	TD	RD	TD	RD	TD
RT	211.5	201.2	256.9	246.9	6.9	5.3
50	189.5	182.7	233.8	222.5	14.6	13.4
75	151.3	136.0	195.0	190.8	15.4	15.3
100	170.9	168.6	178.5	173.3	12.8	11.2
125	130.8	127.3	149.6	141.6	14.7	12.2
150	109.3	96.9	121.3	108.1	15.3	14.5
